# Glyphosate dose modulates the uptake of inorganic phosphate by freshwater cyanobacteria

**DOI:** 10.1007/s10811-017-1231-2

**Published:** 2017-07-21

**Authors:** Damian Drzyzga, Jacek Lipok

**Affiliations:** Faculty of Chemistry, Opole University, Oleska 48, 45-052 Opole, Poland

**Keywords:** Glyphosate, Cyanobacteria, Phosphonate, Phosphorus uptake, Phosphorus binding

## Abstract

The usefulness of glyphosate [*N*-(phosphonomethyl)glycine] as a source of nutritive phosphorus for species of halophilic cyanobacteria has been postulated for years. Our results indicate a stimulating effect of glyphosate on the growth of four out of five examined freshwater species, *Anabaena variabilis* (CCALA 007), *Chroococcus minutus* (CCALA 055), *Fischerella* cf. *maior* (CCALA 067) and *Nostoc* cf. *muscorum* (CCALA 129), in a manner dependent on the applied concentration. The most significant stimulation was observed at a dose of 0.1 mM glyphosate. The decrease in the amount of phosphonate, which correlated with microbial growth, demonstrated that glyphosate may play an important role in cyanobacterial nourishment. Surprisingly, the consumption of organic phosphorus did not start when concentrations of inorganic phosphate (PO_4_^3−^) had fallen dramatically; instead, the assimilation of both types of phosphorus occurred simultaneously. The greatest decrease in the amount of glyphosate was observed during the first week. The uptake of the standard nutrient-phosphate (PO_4_^3−^), was strongly dependent on the xenobiotic concentration. When a concentration of 0.1 mM glyphosate was used, the consumption of phosphate decreased in favour of glyphosate assimilation. Our study revealed for the very first time that the presence of inorganic phosphate significantly enhances the bioavailability of glyphosate. Statistical analysis confirmed that the nutritive usage of glyphosate and the absorption of phosphate are features associated with the herbicide concentration rather than features related to the species of freshwater cyanobacterium. This finding supports the thesis of an important role of organic phosphorus in the formation of cyanobacterial blooms and creates the opportunity of using these cyanobacteria to bind both organic and inorganic forms of phosphorus in microalgal biomasses.

## Introduction

Glyphosate [*N*-(phosphonomethyl) glycine] (NPMG) belongs to group of non-selective herbicides with a broad spectrum of action and is the most commonly used worldwide for weed control (Goldsborough and Brown 1988). Because of its relatively low mammalian toxicity (Rios et al. 2002) and broad range of applications, glyphosate has been the most used pesticide at the global scale for many years, with its price decreasing with each year (Woodburn 2000). The introduction of genetically modified plants resistant to glyphosate (so called “Roundup Ready” (RR), genetically engineered (GE) herbicide-tolerant (HT) plants) has made a great impact on the worldwide consumption of glyphosate in recent years (Coupe et al. 2012). From 1995 to 2014, the worldwide usage of this herbicide (in both agricultural and non-agricultural applications) has risen over 12-fold. The decade from 2004 to 2014 was a period when glyphosate was applied on an unprecedented scale: 6.1 billion kg of glyphosate was used, which constitutes 71.6% of the total use worldwide from 1974 to 2014 (Benbrook 2016). Glyphosate residues are often identified in environmental water samples. In 2002, *N-*phosphonomethyl glycine and AMPA were detected in the Mississippi River Basin at levels above 0.1 μg L^−1^ in 40% of tested streams samples. In 2004 and 2005, glyphosate was detected in 26 and 17% of samples, respectively, at concentrations above 5 μg L^−1^ (Coupe et al. 2012).

The fate of any pesticide in the environment depends on the physicochemical properties of the compound and the soil and meteorological factors (Salmon-Monviola et al. 2011). The high solubility of glyphosate in water (Battaglin et al. 2005) in combination with surface run-off and washout from the ground increases the chance of introducing this xenobiotic to aqueous systems (Tsui and Chu 2008). It has been suggested that glyphosate is an environmentally friendly chemical compound that does not have a negative impact on other organisms, except plants (Williams et al. 2000). Recently, there have been some reports suggesting its toxic effects on some aquatic organisms (Wang et al. 2016). It has been proven that the presence of glyphosate could change the composition of algae communities at a level of 10 μg L^−1^ (Pesce et al. 2009) and could negatively affect some freshwater phytoplankton strains (Vendrell et al. 2009). NPMG exposure can reduce diatom abundance, as well as enhance the development of cyanobacterial colonies (Vera et al. 2010). The presence of glyphosate could also stimulate the growth of some aquatic photoautotrophs. For example, the cyanobacterium *Anabaena variabilis L.* can not only tolerate treatment with NPMG but is also able to decompose this substance and use it as source of phosphorus (Ravi and Balakumar 1998). This ability of cyanobacteria could contribute the its ecological advantage over other organisms and may lead to the formation of harmful blooms (Smith 2003; O’Neil et al. 2012). The problem of cyanobacterial blooms has become more and more serious from ecological point of view, as well as in terms of the economy and protection of human health (Sharpley and Wang 2014; Khan et al. 2015).

Cyanobacteria are known for their outstanding adaptive capabilities (Kasowska-Żok et al. 2014). These microorganisms play a major role in the global cycling of nitrogen and carbon and are the only group of prokaryotes able to carry out the oxygenic photosynthesis. Species that fix atmospheric nitrogen increase its concentration in the soil. Cyanobacteria possess various environmental adaptations, including the ability to obtain all forms of phosphorus from the environment (Tiwari et al. 2015; Lipok et al. 2007). The secretion of enzymes responsible for the assimilation of phosphorus outside the cell is one of the ways to obtain this nutrient (Ravi and Balakumar 1998). Glyphosate, when is introduced into water reservoirs, increases the pool of existing organic phosphorus (Battaglin et al. 2014). It enters the pool of dissolved organic phosphorus (DOP), which together with a variety of forms of inorganic phosphorus (P_i_) can be found in oligotrophic waters (Kretz et al. 2015). Although phosphorus, as a nutritive element, is necessary to sustain the growth of primary producers in aquatic environments (Girault et al. 2012), the issue of which form of this nutrient is appropriate for different aquatic species remains unsettled. For years, many attempts have been made to estimate the contribution of phosphonates to total DOP pool. Currently, it is thought that up to 25% of organic phosphorus in aquatic environments exists in the form of phosphonates; however, it is more likely that this value is approximately 10% (Van Mooy et al. 2015; Lin et al. 2016). Environmental phosphorus redox cycling determines the DOP pool composition and exerts an indirect impact on phytoplankton communities (Lin et al. 2016; Van Mooy et al. 2015; Benitez-Nelson 2015). Thus, it seems obvious the Pi uptake by microorganisms is limited by the concentration of ambient phosphorus.

Phosphorus metabolism has been well-documented for many species of heterotrophic bacteria. However, the dependency on glyphosate and phosphate consumption is still uncertain for prokaryotic autotrophs such as cyanobacteria. Based on the findings of our study, we hereby report the interactions between five cyanobacterial strains and glyphosate in the context of microbial sensitivity towards glyphosate and the impact of this herbicide on the utilization of inorganic phosphate. Furthermore, the effect of the concentration of this xenobiotic, as the crucial factor for its bioavailability to cyanobacteria, was investigated.

## Methods

### Cyanobacterial strains, culturing conditions

Five species of freshwater cyanobacteria were purchased from the Culture Collection of Autotrophic Organisms (CCALA, Institute of Botany, Academy of Sciences of the Czech Republic, Trebon). They are characterized in Table 1. For a better understanding of the impact and bioavailability of glyphosate, the tested species, representing four out of five taxonomic groups (section) of cyanobacteria (Rippka et al. 1979), were selected with respect to the differences in their colony organization, cell shape, mode of cell division, occurrence in nature, and production of toxins (Castenholz and Waterbury 1989).

**Table 1:** Used cyanobacterial species

Strain No	Species	Section	Origin	Habitat
CCALA 007	*Anabaena variabilis*	IV	USA, Mississippi	Freshwater
CCALA 049	*Chroococcidiopsis thermalis*	II	Slovakia, Piestany	Thermal mud
CCALA 055	*Chroococcus minutus*	I	Macedonia, Ohrid	Lake, littoral
CCALA 067	*Fischerella* cf. *maior*	V	Switzerland, Aargau, Mellingen	No available data
CCALA 129	*Nostoc* cf. *muscorum*	IV	Poland, Lublin	Lake

Based on the *CCALA*, Culture Collection of Autotrophic Organisms, data

Because the selected species are representative of each section, this allows for the study of the specificity of dynamic interactions with NPMG and the cellular response of particular organisms, which can then be broadly applied to cyanobacteria in general.

Prior to being grown in experimental media, cyanobacteria were pre-grown in 50 mL of BG11 medium (ATCC 616) (Rippka et al. 1979) in 250-mL Erlenmeyer flasks. Microorganisms were revitalized every 21 days by transferring 10 mL aliquots to 50 mL of fresh media. Cyanobacteria were cultivated at 24 ± 1 °C, and the photoperiod was set at 16 h: 8 h (day: night) at 200 μmol photons m^−2^ s^−1^ PAR. P-depleted conditions were obtained by removing K_2_HPO_4_ from standard BG11 medium (Bg11-P); in this case it was necessary to balance the potassium concentration by providing this element in the form of KNO_3_ (34 mg L^−1^), which resulted in a reduction, the amount of NaNO_3_ to 1.47 g L^−1^ (to keep the amount of N balanced). The same conditions were maintained for supporting the growth of experimental cultures. All chemicals necessary to prepare cyanobacterial media were purchased from POCh (Poland).

### Glyphosate treatments

Pure, powdered glyphosate was obtained by applying a procedure used in our laboratory that has been described previously (Lipok et al. 2010). To maintain sterile conditions, appropriate amounts of each tested phosphonate were dissolved in a few millilitres of sterile medium and were added to the final solutions via filtration through sterile membrane syringe filters (Nylon, 0.22 μm).

Tested cyanobacterial strains were cultured for 14 days in BG11 medium supplemented with the following concentrations of glyphosate: 0.05, 0.1 and 0.2 mM. The range of applied concentrations was based on previous results (data not shown), and it covers the herbicide doses that do not negatively influence the growth of the majority of examined freshwater cyanobacteria. Concentrations of NPMG above 0.2 mM led to culture dieback over 2 weeks. In all systems, the initial chlorophyll concentration was set at 1 mg L^−1^. The PO_4_^3−^ concentration was the same at the start of all tests - 0.03 g L^−1^ of K_2_HPO_4_. Control cultures were not supplemented with the tested phosphonate. Abiotic controls to assess glyphosate stability and its tendency to adsorb on glassware used in this experiment were also performed. Briefly, sterile 0.1 mM solutions of glyphosate in Bg11 medium specifically formulated for cyanobacteria were maintained at the same light, temperature and humidity conditions as used for the experimental cultures. The concentration of NPMG at the beginning and at end of the experiment was determined via high-performance liquid chromatography (HPLC). All experiments were carried out in triplicate (*n* = 3).

### The evaluation of the growth of cyanobacteria based on the content of chlorophyll

Determination of cyanobacterial growth based on measurements of total chlorophyll content over time in the experimental cultures was performed according to the method described previously (Lipok et al. 2010). Briefly, at 3–4-day intervals, growth measurements were made in harvested samples as follows: (i) 1-mL samples were taken from each repetition, and (ii) the cells were sedimented via centrifugation for 5 min at 13000*×g*, (iii) obtained pellets were resuspended in 0.9 mL of methanol, and solubilization was allowed to proceed for 20 min in the dark, with occasional mixing, and then (iv), step ii was repeated. Total chlorophyll content in the supernatant was determined spectrophotometrically based on Arnon’s formula: Total chlorophyll [*a* + *b*] = 20.21 A_645_ + 8.02 A_663_ (Porra 2002).

### Quantitative determination of phosphate and phosphonate levels in cyanobacterial cultures

Media harvested during the experiments were separated from the cells via centrifugation at 13,000*×g* for 5 min. Then, the cell-free supernatants were frozen and were kept at −28 °C until the day of analysis.

^31^P NMR (nuclear magnetic resonance) spectroscopy was carried out to estimate the amount of inorganic phosphate. Samples (450 μL) of media were placed in NMR cuvettes (5 mm in diameter) and mixed with 123 μL of a 0.01 M solution of EDTA. ^31^P NMR experiments were performed using a Bruker Avance DRX 400 spectrometer operating at 161.976 MHz. Data were acquired at 20 ± 1 °C using a 30° pulse, a 1.337 s acquisition time and a 0.5-s relaxation delay, with a 20 Hz spin rate (5-mm probe). The number of scans was 900, with an FID resolution of 0.374 Hz. The 0.01 M solution of H_3_PO_4_ was used as the internal reference standard. The concentration of phosphate in these samples was estimated by integrating the area of its signal with respect to that of the internal reference standard using MestReNova version 6.0.2 software.

The amount of NPMG was estimated based on the results of the HPLC measurements. Pre-column derivatization with *p*-toluenesulphonyl chloride was carried out to quantify residual glyphosate levels in post-culture media according to a procedure described previously (Khrolenko and Wieczorek 2005). After the derivatization process, according to a previously described method (Lipok et al. 2010), the samples were injected onto a 4.6 mm × 250 mm Phenomenex Kinetex Evo C-18 column. The mobile phase was composed of 0.01 M KH_2_PO_4_ (pH 2.3) and MeCN (90:10, *v*/v). The flow rate for isocratic separation was adjusted to 1 mL min^−1^, with the eluate monitored at 240 nm. Each sample was measured in triplicate. Concentrations of glyphosate were computed based on calibration curves established using a stock solution of NPMG in Bg11 medium.

Agglomerative hierarchical clustering (AHC) was performed using XLSTAT version 2014.5.03. Dissimilarity was described by the Gower coefficient, and the unweighted pair-group average agglomerative method was used.

### Estimation of generation (doubling) times

Generation (doubling) times (DTs) of the tested cyanobacteria were calculated mathematically. The growth curve was fitted by applying the exponential growth eq. *Y* = *Y*_0_ e^(k*x)^, where *k* is the growth rate constant, expressed as a reciprocal of the time units, and *x* is time. Therefore, generation time (DT) was computed as ln(2)/k. For each replication, the DT was calculated separately, and hence, this value is presented as the mean ± S.D. (*n* = 3). Computations were performed using GraphPad Prism version 5.01.

## Results

### Glyphosate effectively stimulates cyanobacterial growth

**Fig. 1:**
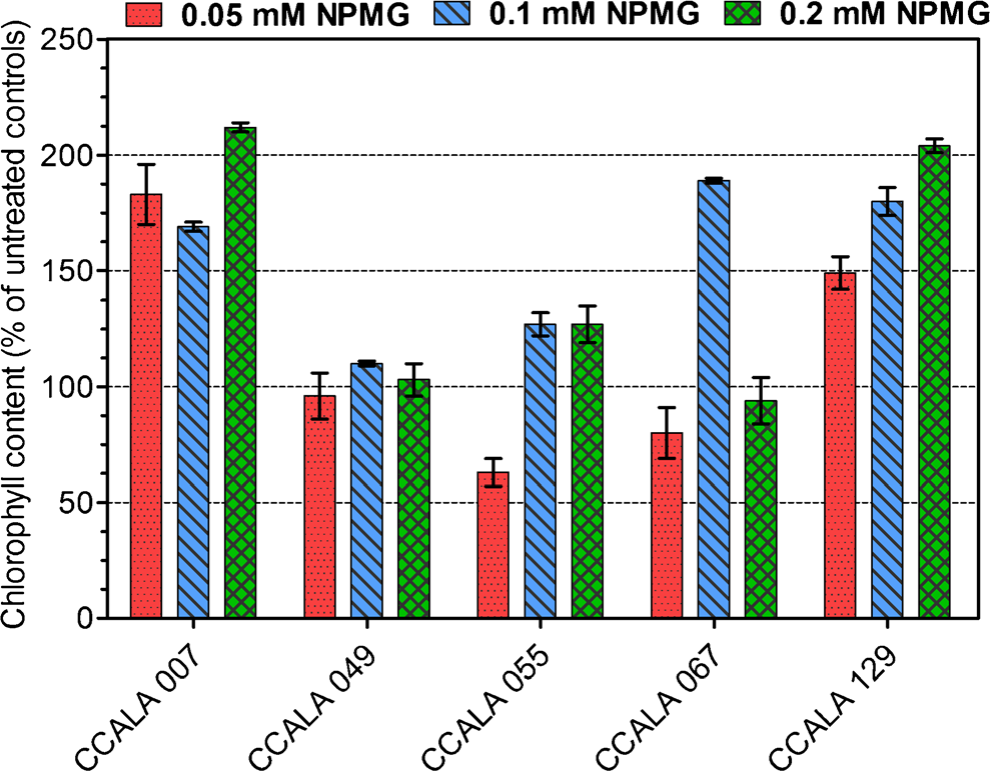
Effect of glyphosate treatment on cyanobacterial growth after 2 weeks. Results are given as a percentage growth relative to untreated controls (%) with ± S.D. for three replicates

The concentration of chlorophyll in cyanobacterial cells was the main factor taken in to account in evaluating the impact of glyphosate on the growth of bacteria. Significant stimulation was noticed for the four tested species treated with 0.1 and 0.2 mM doses of NPMG (Fig. 1), with CCALA 007 (hereafter, *Anabaena* 007) and CCALA 129 (*Nostoc* 129) cultures containing almost twofold higher concentrations of chlorophyll than their respective controls. Interestingly, the growth of both strains was supported similarly by the lowest tested glyphosate dose −0.05 mM.

A dose of 0.1 mM glyphosate was the only concentration that stimulated (almost twofold increase) the growth of *Fischerella maior* (CCALA 067), and similar to the effect of the 0.2 mM dose, it induced an approximately 30% increase in the content of chlorophyll in *Chroococcus minutus* (CCALA 055) cultures. Glyphosate neither hampered nor supported the growth of *Chroococcidiopsis thermalis* (CCALA 049). The stimulation of microbial growth could be explained as an effect resulting from one or both of the following phenomena: (i) the intensification of inorganic phosphate uptake and (ii) the acquisition of nutritive phosphorus from glyphosate.

### Utilization of inorganic phosphate in relation to glyphosate concentration

The co-existence of two studied forms of phosphorus (Pi and NPMG) in the environment suggested the importance of simultaneously assessing the consumption of inorganic phosphate, represented as the amount of residual phosphate present during the growth of cyanobacteria and the utilization of glyphosate, which was expressed based on the extent of its decrease over the course of the experiments.

**Fig. 2:**
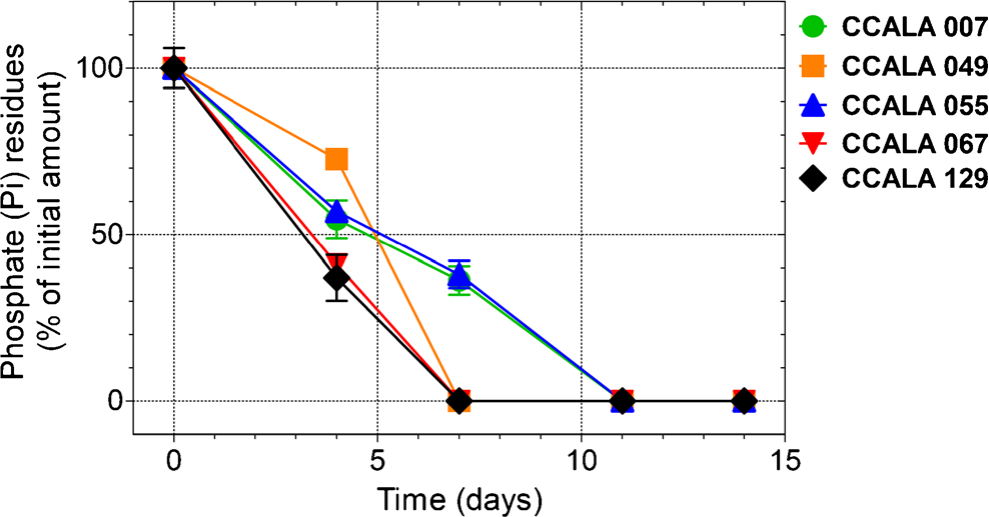
Percentage decrease of phosphate content in cultures of cyanobacteria not treated with glyphosate (controls). Measurements were performed using NMR spectroscopy; thus, the baseline value should be regarded as the concentration below the limit of detection (80 μM in this study) and does not necessarily imply a total lack of phosphate. For each species, the values were calculated separately ± S.D. for three replicates

**Fig. 3:**
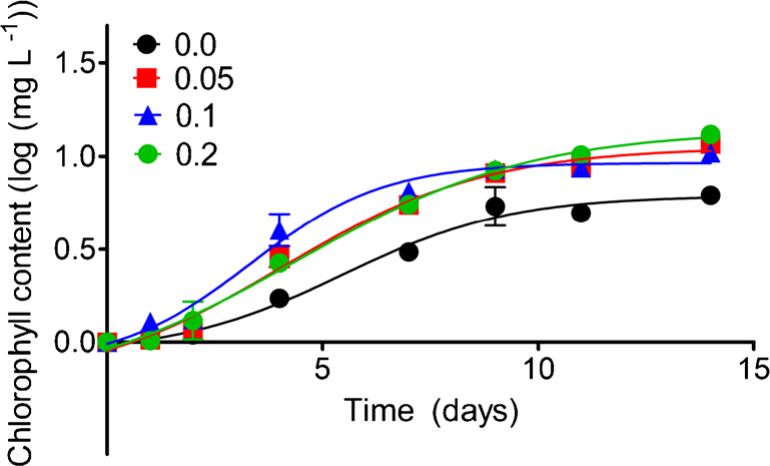
Time course of *Anabaena* 007 growth in the presence of various concentration of NPMG; phosphonate was added to the standard BG11 medium. Growth was measured as the increase of chlorophyll content, and data were related to time (days). Each point is the mean ± S.D. for three replicates

The results for cyanobacterial colonies not treated with glyphosate (controls) (Fig. 2.) revealed a high-level of phosphate decline during the first week. The exceptions were *Anabaena* 007 and *Chroococcus* 055, for which the decrease of P_i_ was slower and continued into the eleventh day. The disappearance of phosphorus, an essential nutrient for intensive cellular metabolism (Garcia et al. 2016), coincided in time (first week of treatment) with a doubling of the growth of cells (Table 2. and Fig. 3.). The dynamics of the growth of *Anabaena* 007 cultures, with a representative plot shown in Fig. 3, clearly demonstrates the stimulating effect of the applied glyphosate.

**Table 2:** Generation times (DTs) of examined cyanobacteria in relation to the concentration of glyphosate added to the medium

Strain	Concentration of NPMG (mM)			
	0.0	0.05	0.1	0.2
	Generation (doubling) time (days)			
*Anabaena* 007	3.8 ± 0.3	2.7 ± 0.3	2.8 ± 0.1	2.6 ± 0.1
*Chroococcidiopsis* 049	4.1 ± 0.7	3.5 ± 0.5	3.4 ± 0.4	4.4 ± 0.4
*Chroococcus* 055	4.5 ± 0.3	3.3 ± 0.3	3.0 ± 0.1	2.9 ± 0.4
*Fischerella* 067	5.5 ± 1.0	6.1 ± 2.5	4.1 ± 0.3	5.1 ± 0.1
*Nostoc* 129	11.0 ± 0.1	8.4 ± 0.7	5.7 ± 0.1	5.3 ± 0.4

Generation times, given in days, were calculated for the first week of cyanobacterial growth in the presence of various concentration of glyphosate (mM). The results are presented as the means ± S.D. for three replicates. When NPMG stimulated cyanobacterial growth, the DT was shorter than for the appropriate control

**Fig. 4:**
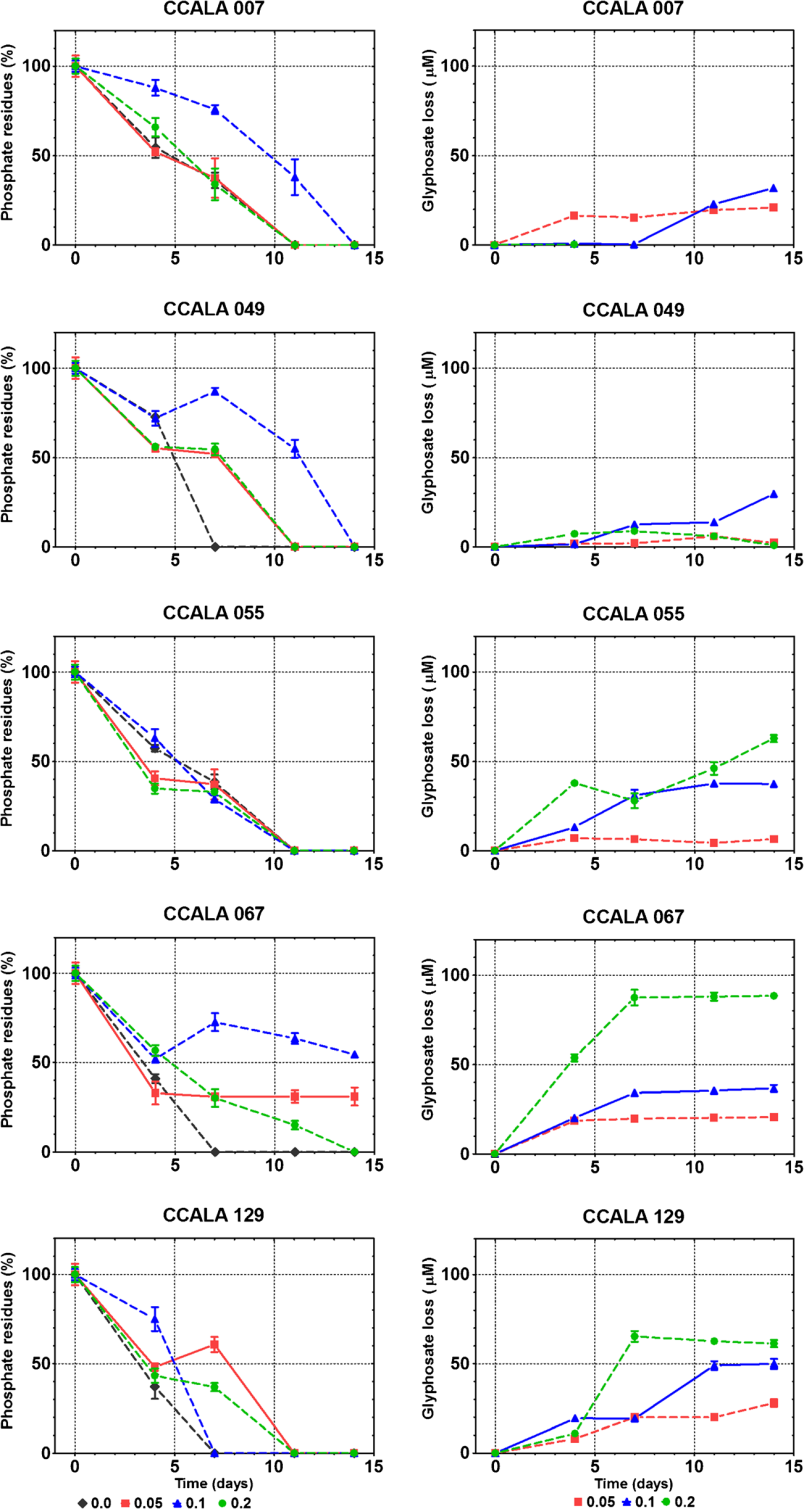
Impact of glyphosate on inorganic phosphorus (phosphates) uptake (*left column*), and the changes in its concentration resulting from the response of cyanobacterial cells (*right column*). The amount of residual phosphate in the experimental media (*left column*) was measured using ^31^P NMR spectroscopy, with 0.01 M phosphoric (V) acid used as an internal standard. The calculated values were compared with those from day 0 and expressed as a percent (%) ± S.D. for three replicates. The amount of NPMG on the following days of the experiment (*right column*) was determined based on HPLC analysis of post-culture media. The disappearance of glyphosate is expressed in μmol L^−1^ ± S.D. for three replicates

According to our findings, glyphosate influenced phosphate uptake by cyanobacterial cells in a dose-dependent manner (Fig. 4.). Tracking concentrations of phosphate, a reduction in the absorption of inorganic forms of phosphorus was observed, which appeared mostly at a concentration of 0.1 mM glyphosate. This dose of NPMG resulted in colonies of *Anabaena* 007, *Chroococcidiopsis* 049 and *Fischerella* 067 limiting or even ceasing the use of phosphate, which was detected up to the very end of experiment. This finding strongly demonstrates the relationship between phosphonate and phosphate in phosphorus use by cyanobacteria. Only in the case of *Chroococcus* 055 did the uptake of phosphate appeared to be uninfluenced by glyphosate. Between the fourth and seventh day of subculturing with 0.1 mM NPMG for *Chroococcidiopsis* 049 and *Fischerella* 067 and with 0.05 mM glyphosate for *Nostoc* 129, a significant increase in the total amount of available inorganic phosphate in the medium was noticed (Fig. 4.). This finding, along with the simultaneous rapid growth of cyanobacteria, indicates the assimilation of glyphosate.

### Assimilation of glyphosate is a dose-dependent process

Since no statistically significant changes were observed in the concentration glyphosate alone, which maintained a stable (100 ± 5%) level in the BG11 medium (substrate control) during 2 weeks of incubation, the decrease in its amount in the experimental treatments resulted from microbial consumption. The dose-dependent assimilation of glyphosate by microorganisms (expressed as its diminishing concentration in culture media) was investigated thorough HPLC analysis of post-culture media (Fig. 4.). For the majority of the tested strains, a significant loss of glyphosate was detected between the fourth/seventh and the eleventh day of the experiment, which coincided with the period of logarithmic cyanobacterial growth and the dominance of the second generation of cells (Table 2.).

NPMG reductions were found to be dependent on its concentration in the medium and on the studied strain. When the tested compound was present at 0.2 mM, a greater reduction was observed. In *Fischerella* 067 cultures, a loss of over 42% (equivalent to an ~90 μM change) of glyphosate was observed, whereas for *Chroococcus* 055 and *Nostoc* 129, the decrease in glyphosate was slightly above 25% of its initial amount. The same concentration of NPMG (0.2 mM) seemed to be unavailable to *Anabaena* 007 and *Chroococcidiopsis* 049.

The fact that DTs for most species were less than 5 days (Table 2) indicates that is was primarily the second generation of cyanobacterial cells that were responsible for the use of glyphosate. This process was especially effective in media initially containing 100 μM NPMG, where within 14 days, all five species of tested cyanobacteria had reduced the concentration by over 30 μM, with *Nostoc* 129 reducing it by 50 μM.

**Fig. 5:**
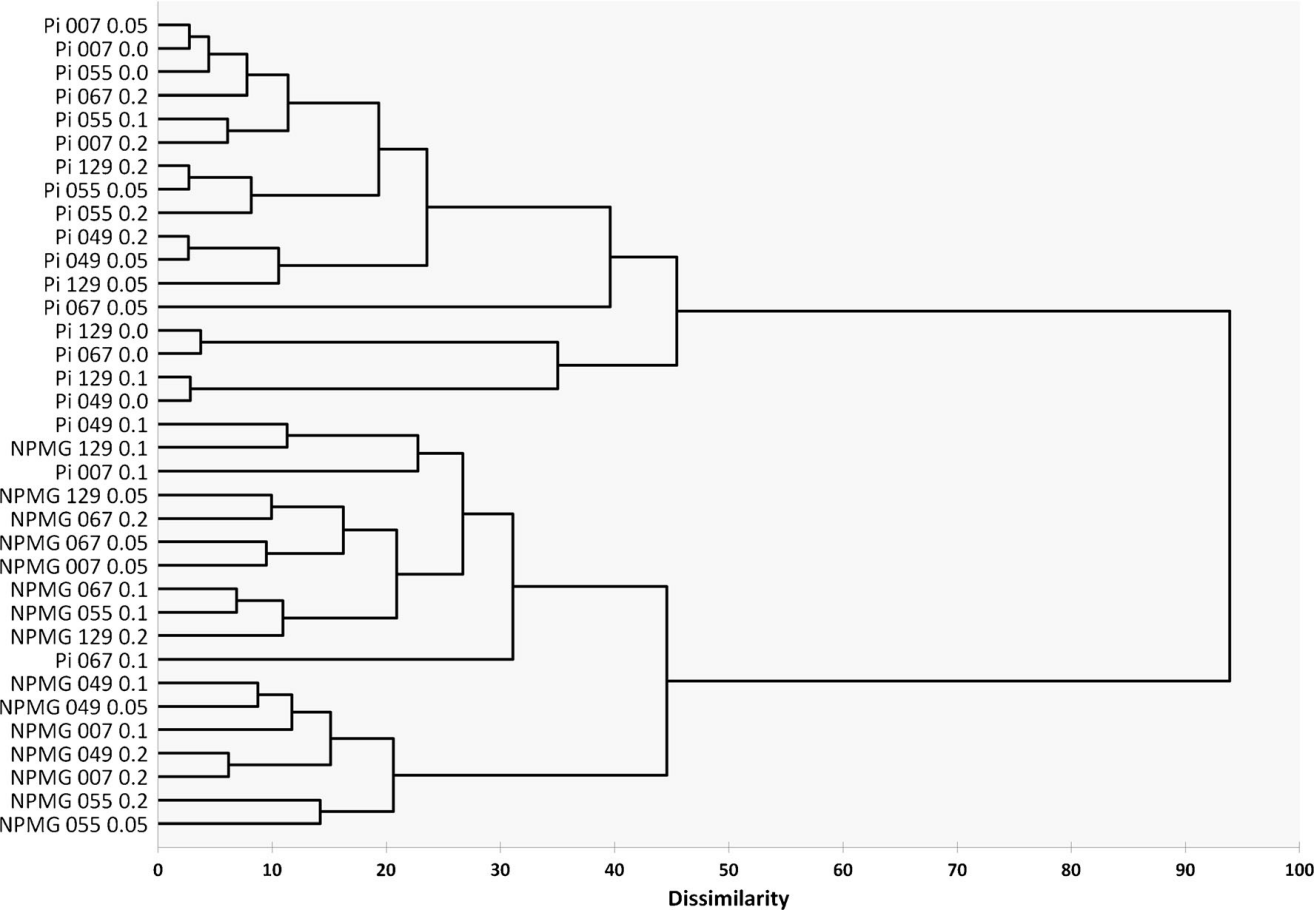
Agglomerative hierarchical clustering (AHC) for residual amounts of inorganic phosphate and glyphosate in cyanobacterial cultures in the presence of various phosphonate concentrations until the 11th day (Gower coefficients with the agglomeration method: unweighted pair-group average). Pi residual inorganic phosphate, NPMG residual phosphonate

AHC allowed the identification of the cyanobacterial response to the presence of NPMG for certain classes of organisms (Fig. 5), which opened the way to understanding glyphosate’s influence on the microbial strategy towards phosphorus consumption. The primary aim of clustering was to determine which of the tested microorganisms use similar strategies when these two different forms of phosphorus are offered. The presence of two separate clusters, one grouping the experimental results in relation to the consumption of inorganic phosphorus and the second containing mainly the results reflecting the reduction in glyphosate, indicate that with respect to the amount of each form of nutritive phosphorus available in the medium, the examined strains used different strategies. Moreover, a deeper analysis of the clustering results, combined with the quantification of phosphorus consumption, proved that glyphosate affects PO_4_^3−^ uptake and that this impact depended on the concentration of glyphosate added. For *Anabaena* 007, 0.05 mM NPMG did not affect phosphate assimilation when compared to the control. Nonetheless, in colonies of *Fischerella* 067 and *Nostoc* 129, the addition of 0.05 mM glyphosate dramatically changed the manner of P_i_ consumption with respect to untreated cells. The fact that glyphosate at a concentration of 0.1 mM interacts with phosphate uptake in a manner dependent on the strategy of the a certain strain was clearly shown for three species: *Fischerella* 067, *Chroococcidiopsis* 049 and *Anabaena* 007, which showed the highest dissimilarity compared to the rest of the strains.

In the light of these results, the disappearance of phosphate from the growth medium was not solely dependent on the concentration of glyphosate. Interestingly, the decrease in glyphosate seemed to also strongly depend on the appropriate concentration of NPMG. Different concentrations, such as 0.05 mM for *Fischerella* 067 and *Anabaena* 007, 0.1 mM for *Fischerella* 067 and *Chroococcus* 055, and the highest concentration of 0.2 mM for *Anabaena* 007 and *Chroococcidiopsis* 049, resulted in groups showing similar responses.

### Glyphosate utilization is enhanced by inorganic phosphate

**Fig. 6:**
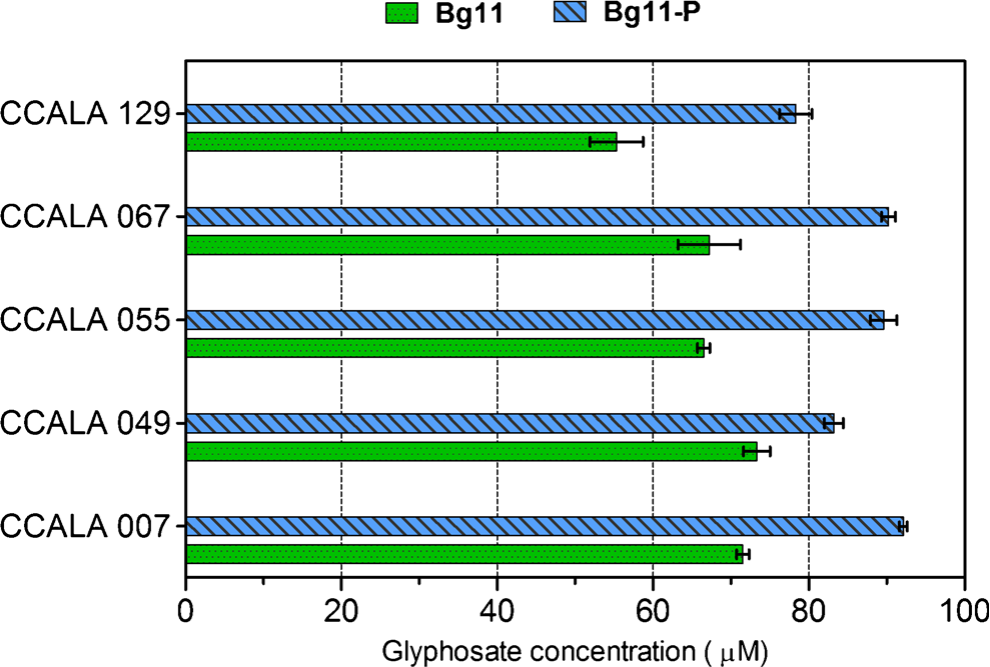
Glyphosate concentration in cyanobacterial cultures after 2 weeks. In all cases, the initial amount of NPMG was 100 μM; however, in BG11 media, this substance was an additional source of phosphorus along with Pi, while in BG11-P, it was the sole available source of this element. The results are given ± S.D. for three replicates

When glyphosate (in the dose 0.1 mM) was the sole phosphorus source in media, the tested cyanobacteria did not exhibit satisfactory growth during the 2 weeks of the experiment (data not published), and during this time, the concentrations of this substance decreased only moderately. The HPLC analysis of cyanobacterial post-culture media revealed that in addition to glyphosate affecting phosphate assimilation in a dose-dependent manner, the presence of inorganic phosphate also influences NPMG availability (Fig. 6.). The results obtained from both BG11 and BG11-P media show that the reduction in glyphosate concentration after 2 weeks in Pi-depleted conditions was less by approximately 20 μM. It can be concluded from this that a mutually dependent impact of both form of phosphorus was observed in all experiments.

## Discussion

This study showed that the uptake of inorganic phosphate (PO_4_^3−^) by freshwater cyanobacteria, which had grown in the presence of glyphosate, was correlated with the provided dose of this herbicide. Thus, the supplementation of media with glyphosate affected the uptake of PO_4_^3−^ by the cells. Some of following cellular response strategies have been predicted: (1) increase or (2) decrease in the rate of P_i_ consumption, (3) maintaining consumption at the same level, or (4) discontinuation of the use of inorganic phosphorus. However, the last response seemed to be almost improbable in the context of the proven bioavailability of phosphates and the importance of phosphorus for the development of bacterial colonies (Schweitzer and Simon 1995; Toolan et al. 1991).

For colonies not treated with NPMG, inorganic phosphorus loss was highest in the first seven (or 11) days of culturing, which coincides with the logarithmic phase of cyanobacterial growth. Obviously, such a high demand is related to many aspects of the regulation of cellular processes, including the phosphorylation of proteins and biosynthesis of intracellular components, because phosphorus-containing compounds are involved in many metabolic pathways (Dick et al. 2011; Wu et al. 2003; Heath et al. 2016). It is also known that phosphorus limitations can directly affect the composition of bacterial cell walls (Liu et al. 2016). Therefore, the demand for phosphorus is correlated with an attempt to maintain phosphorus homeostasis in cyanobacterial cells.

Results of experiments with glyphosate have shown that its presence affects the rate of microbial consumption of inorganic phosphorus. Decreasing concentrations of NPMG together with a growth rate of experimental colonies that exceed that of their respective controls undoubtedly demonstrates the importance of this phosphonate as a nutrient fully equivalent to PO_4_^3−^. The exception was *Chroococcus* 055; in this case, Pi management was not affected by glyphosate. Conceivably, the reason for this was the cellular organization of the colony because this species is the only one among of all of the examined strains that forms colonies composed of two, four, or more cells sheltered by a transparent sheath constructed mainly of polysaccharides. Such a natural barrier usually limits the migration of elements/nutrients to inside the colony (Song et al. 2015).

Glyphosate, when tested in the applied doses, did not show any lethal impact on freshwater cyanobacteria. Furthermore, the growth-supporting effect was enhanced with increasing doses of NPMG. This was expected because it has already been proven for the halophilic cyanobacterium *Spirulina platensis* (Lipok et al. 2007). Hence, phosphorus-containing xenobiotics may serve as a useful source of nutritive phosphorus, and the intensity of their uptake depends more on their concentration than on the taxonomic classification of the species. Our results show that glyphosate is utilized by cyanobacteria mainly in the logarithmic phase of their growth, when its use prevails over that of phosphate, which is used more in the lag phase. Therefore, the presence of glyphosate at appropriate concentrations is a highly significant factor for the phosphorus utilization strategy of cyanobacteria. It has been postulated that phosphorus uptake from the DOP pool may dominate over Pi uptake but only when the available DOP is in excess (Cotner and Wetzel 1992). In our study, this relation was maintained only for the highest applied concentration of NPMG, 0.2 mM, which was equivalent to 6.20 mg L^−1^ of P. Moreover, in this case, the amounts of DOP and dissolved inorganic phosphorus-DIP (DIP was always present at an initial concentration of 5.34 mg L^−1^ P) were very close to each other. Therefore, the utilization of NPMG, especially when it was tested at lower concentrations, occurred in a more specific manner. The possibility that the consumption of glyphosate, which was the only form of DOP present, was associated with the secretion of enzymes responsible for the acquisition of phosphorus from organic molecules should not be excluded (Singh et al. 2006). It has been proven that 5 days of culturing in the presence of glyphosate evokes an increase in the concentration of extracellular proteins and carbohydrates, as was demonstrated for several species of green algae: *Scenedesmus quadricauda, Chlorella kessleri* and *Raphidocelis subcapitata* (known as *Selenastrum capricornutum*) (Maršálek 1996).

It was predicted that unfavourable growth conditions resulting from phosphate (Pi) depletion in media would intensify NPMG uptake due to the necessity of supplying this essential nutrient. Instead, our study, for the very first time, revealed that it was the presence of inorganic phosphate that significantly enhanced the bioavailability of glyphosate. It is thought that in millimolar concentrations, glyphosate diffuses through membranes. In contrast, at micromolar levels, the transportation of this substance is accomplished by high affinity phosphate transporters that show a low affinity towards the phosphonate (Hetherington et al. 1998). Such transportation would result in only small amounts of NPMG being absorbed by cells. The regulation of Pi transport in microorganisms is often under Pho (phosphate) regulon control, which could sense the environmental concentration of Pi and, if phosphate appears to be limited, influence the expression of relevant genes. This up- and down-regulatory system in bacteria is composed of the protein pair PhoB–PhoR (Tommassen et al. 1982), which in the case of cyanobacteria is under the control of the histidine kinase-response regulator pair SphS–SphR (Juntarajumnong et al. 2007). Extracellular levels of Pi are detected by the PstSCAB complex (*P* *hosphate* *S* *pecific* *T* *ransporter*), which in conditions of surplus phosphate keeps the whole system inactive. When extracellular concentrations of Pi are below 4 μM, a conformational changes in the PstSCAB complex occurs, leading to the phosphorylation of the transcription regulator PhoB, which after dissociation from PhoR binds to the appropriate DNA region (Makino et al. 1988; Lamarche et al. 2008). This sequence of events results in gene expression and the activation of many hydrolytic enzymes (phosphatases) involved in DOP utilization (Santos-Beneit 2015). It is hard to predict whether all of these phenomena took place in our experiments; nevertheless, the reduction in glyphosate content in experimental media seems to confirm the occurrence of these processes.

It has been proved that cyanobacteria play a major role in nitrogen, carbon and phosphorus cycling in nature (Forlani et al. 2015). However, the strains that utilize aminophosphonates seem using these compounds as nutrients, rather as a source of phosphorus, than nitrogen. Such a thesis is supported from studies on the availability of both: phosphorus and nitrogen for *S. platensis*, which demonstrated that inorganic nitrogen contained in medium in the form of sodium nitrate, was definitely more preferable, than nitrogen in the form of aminophosphonate (Forlani et al. 2011). In regard to our experiments, applied media: BG11 and BG11-P contained standard nitrogen source—sodium nitrate at the concentration of 1.5 g L^−1^, and such a surplus of nitrogen, in highly preferable form of NaNO_3_, makes glyphosate unlikely to be a N-source per se. Moreover, in our study, we examined diazotrophic species with capacity to assimilate atmospheric N_2_. Thus, in our opinion, cyanobacterial strategy to use phosphonate as nitrogen source instead of NaNO_3_, appears unlikely. Much earlier, in 1993 Martensson tried to assess whether the nitrogen assimilation expressed as nitrogen fixing activity in cyanobacteria could be inhibited by glyphosate addition, and she did not found any remarkable differences between controls and organisms treated by glyphosate (Martensson 1993). From the other hand, in 2016, Bodkhe and Tarar found that for six cyanobacterial strains, low doses of glyphosate even stimulated fixation of N_2_ (Bodkhe and Tarar 2016). Therefore, it cannot be excluded that the management of nitrogen in the cells of cyanobacteria might be affected by glyphosate, but the specific action is still unclear and it is worth further elucidation.

Glyphosate can be decomposed by many bacterial strains (Cook et al. 1978; Lipok et al. 2007; Bujacz et al. 1995; Gomez-Garcia et al. 2011; Dyhrman et al. 2006; Singh 2009; Ford et al. 2010; Sviridov et al. 2015; Zhao et al. 2015). It has been proven that the presence of heterotrophic bacteria in cultures is sometimes connected to the ability of cyanobacteria to degrade organic compounds, indicating that not just cyanobacteria themselves are responsible for such activity (Abed and Köster 2005). However, the utilization of glyphosate presented in our study, as well as its impact on Pi uptake, seems unlikely to be related to the actions of heterotrophic bacteria on a significant scale. Continuous control of the axenic purity of the experimental cultures through microscopic observations strongly suggests that the contribution of cyanobacteria to DOP and DIP mineralization in the environment seems unquestioned. Nevertheless, phosphate metabolism in cyanobacteria is a complex issue because it has been proven that organisms belonging to the same genus can decompose the same substance in completely different ways (Gomez-Garcia et al. 2011). Available sequenced genomes of cyanobacteria are still not complete; therefore, the hydrolytic action of many enzymes related to the Pho regulon (e.g., C-P lyase) is based on hypothetical suppositions.

It is worth emphasizing that the results of our study indicate that the most popular herbicide worldwide may benefit cyanobacteria by providing a source of nutritive phosphorus that is as beneficial as inorganic phosphate. This finding, when correlated with the data on the presence of NPMG in surface waters, supports the thesis of an important role of organic phosphorus in cyanobacterial blooms. Moreover, it creates the opportunity of using cyanobacteria to bind excessive amounts of both organic and inorganic forms of phosphorus in microalgal biomasses.
